# Modified tibiofibular synostosis vs. Burgess stump in a bilateral amputee: a case report

**DOI:** 10.1186/s12891-026-10339-5

**Published:** 2026-10-01

**Authors:** Marc Ruewe, Katharina Rosengarth, Alexandra M. Anker, Sebastian Geis, Luis Sturz, Lukas Prantl, Claudia Fellner, Daniel Deuter, Sebastian Frank, Christina Wendl, Silvan M. Eisenmann

**Affiliations:** 1 Center for Plastic, Hand and Reconstructive Surgery, University Hospital Regensburg, Regensburg, Germany; 2 Department of Neurosurgery, University Hospital Regensburg, Regensburg, Germany; 3 Department of Radiology, University Hospital Regensburg, Regensburg, Germany; 4 Institute for Experimental Psychology, University Regensburg, Regensburg, Germany; 5 Department of Neuroradiology, University Regensburg, Regensburg, Germany; 6 Center for Plastic, Hand and Reconstructive Surgery, University Hospital Regensburg, Regensburg, Mail, Germany

**Keywords:** Transtibial amputation Ertl stump, Tibiofibular synostosis, Myoplasty, Proprioceptive feedback

## Abstract

Despite trans-tibial amputation being the most common major amputation, consensus on optimal stump management remains limited. Cohort studies comparing Ertl-tibiofibular synostosis (TFS) and Burgess-type stumps (BTS) have yielded conflicting results. We present the case of a bilateral trans-tibial amputee with a modified TFS on the right and a conventional BTS on the left. Instead of traditional myodesis, a myoplastic sling was created attaching residual tendons distal to the bone bridge to improve tendon sliding and perceptive feedback. Outcomes were assessed via load distribution, temporospatial gait analysis, adaptability tests, postural control studies, the Four Square Step Test (FSST), stair ascent, functional MRI, and tractography. The TFS stump appeared to show greater ambulation efficiency and was preferred during prolonged standing. FSST and circular gait tests revealed unsteadiness on the BTS side. No activated voxels appeared in the left postcentral gyrus during BTS loading. Movement of the TFS stump activated larger clusters in the contralateral precentral gyrus, while BTS movement activated the supplementary motor area. Fiber connections to the cerebellum via the superior cerebellar peduncle yielded more extensive streamline reconstructions during BTS movement. These unique trial conditions highlight subtle functional differences that are often missed in cohort studies but may significantly impact individual patient needs.

**Supplementary Information:**

The online version contains supplementary material available at https://doi.org/10.1186/s12891-026-10339-5.

## Introduction

**Fig. 1:**
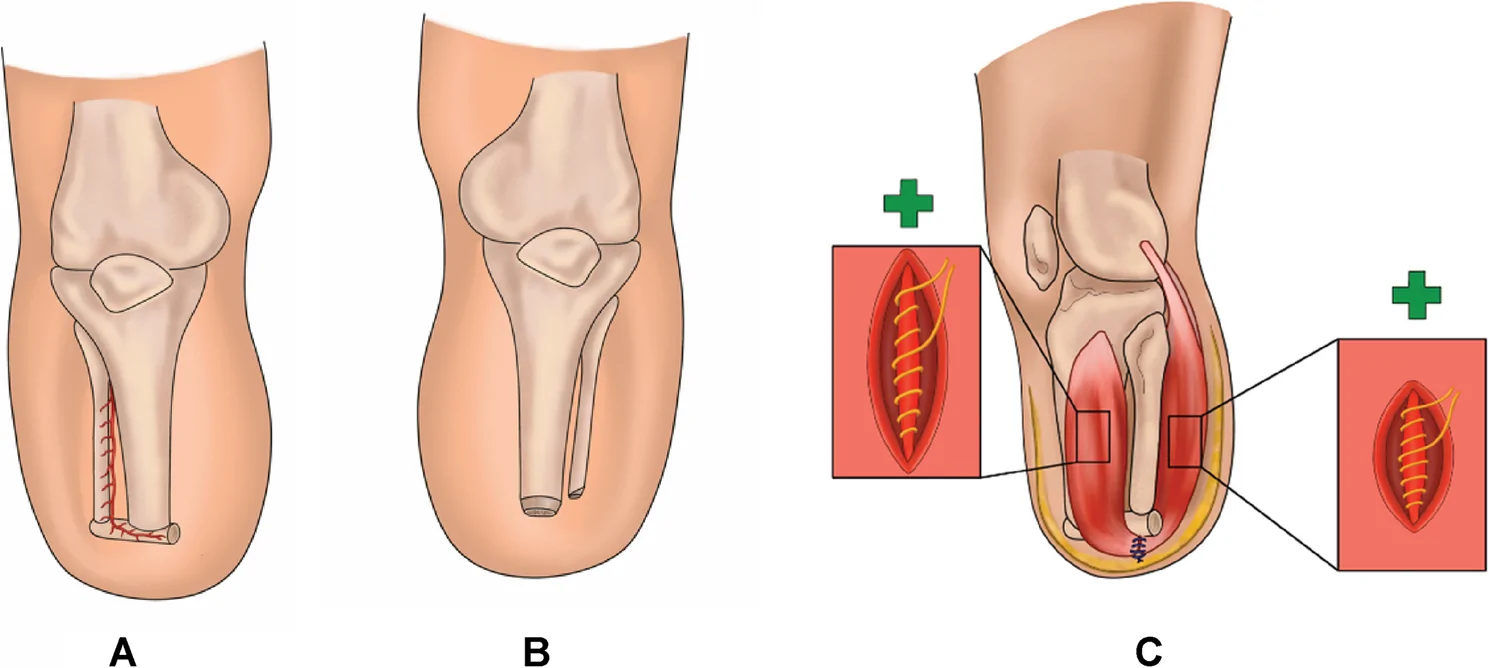
The illustration shows the different bony configurations of the stumps A Ertl stump (TFS), B Burgess stump (BTS), C TFS with tendon sling. **A** The right extremity was managed by tibiofibular synostosis (TFS) with a pedicled fibula bone flap. The stump configuration resembles the anatomy of the original ankle mortice. **B** The left leg necessitated a Burgess type stump (BTS) management. The anterior triangular ridge of the tibia was flattened by osteotomy. **C** For the TFS a myoplasty sling was created by attaching the residual Achilles tendon to the remnant of the tibialis anterior muscle tendon distal to the bone bridge with adequate preload tension. This procedure requires a slightly longer total stump length to preserve the myotendinous portion of the respective muscles. Dynamic muscle afferents from intrafusal muscle fibers and tendons encode the length of individual muscles as well as the speed of stretching to maintain posture and stable gait. Extensor contraction induces stretching and firing of flexor muscle spindles to provide proprioceptive information for equilibrioception

To date more than 378,000 trans-tibial amputees exist in the Unites States (US) only [1, 2]. Demographic changes along with increasing metabolic disease prevalence and related complications, may lead to an estimated number of 3.6 million US-Americans living with the loss of a limb by 2050 [2]. Despite being by far the most frequently performed major amputation type, there is relatively little consensus with regard to optimal surgical stump management in trans-tibial amputees [1, 3, 4]. In fact, amputation procedures are still widely seen as the simple termination of a previous failed limb salvage attempt, as opposed to a reconstructive challenge to perpetuate optimal function of the residual extremity [5]. However, Johann von Ertl was the pioneer to advocate the formation of an osseous bridge between tibia and fibula stumps, to preserve an end-weight bearing extremity [6] (Fig. 1a). Although, this meritorious idea has been published in the late forties of the last century, tibiofibular synostosis (TFS) has rarely been practiced in the meanwhile; thus Ertl-amputees remained an exclusive club ever since. Nevertheless, a small number of trials attempted to compare the Ertl procedure with the Burgess-type stump (BTS) with regard to functional outcome and came to discordant conclusions [4, 7–10] (Fig. 1b). This uncertainty impedes patient counseling with regard to potential advantages and disadvantages of TFS towards the conventional BTS amputation.

However, there is a high chance for inconclusive results, as most cohorts in this field comprise a wide array of different amputation levels, underlying disease conditions (i.e. traumatic vs. degenerative cause of amputation), comorbidities (i.e. phantom limb pain), patient ages and related patient demands and prosthesis provisions. In this respect, research and practice may sometimes appear in disagreement [1].

To our knowledge there has been no report on the clinical outcome of a patient, that underwent bilateral trans-tibial amputation with these two different approaches on either side. Certainly, a single case may not ultimately substantiate the superiority of one or the other procedure, however these very unique trial circumstances may allow the detection of minor distinctions that are otherwise lost in data noise – despite still being perceived as highly relevant for individual patient demands.

### Case presentation

A 48 year old patient was initially submitted to intensive care with the diagnosis of pneumonia associated sepsis. Septic shock conditions indicated high dose i.v. catecholamine therapy with corresponding progressive acral ischemia and subsequent bilateral acral necrosis of both feet. Following recovery from the critical illness phase, severe tissue necrosis indicated trans-tibial amputation of the right side. At this time, necrosis on the left side was managed by amputation in the tarsometatarsal joint line (i.e. Lisfranc level). After hospital discharge, persisting ulcers with exposed tibia and fibula indicated the need for trans-tibial amputation on the left side six months later.

## Methods

### Ethical approval

All procedures were performed in accordance with the relevant guidelines and regulations. Ethical approval for this publication was obtained from the institutional review board of the University Hospital Regensburg (Ref. 22-3031-101).

### Stump management

**Fig. 2:**
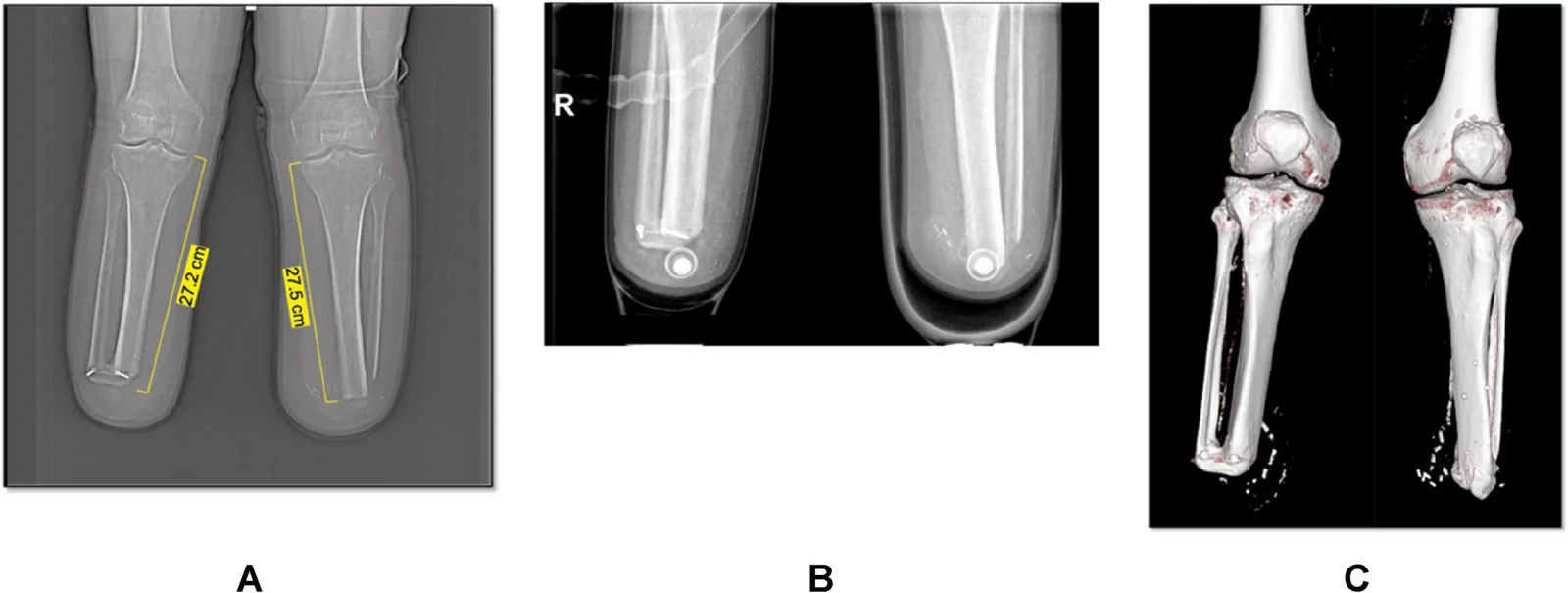
Imaging A X-Ray, B X-Ray during Stance, C 3D Reconstruction of CT-Scan. **A** Anterior-posterior view of the BTS (left) and TFS (right) stumps. At the patient’s request, the stump lengths were intentionally matched. The right stump measured 27.2 cm, while the left tibial and fibular stump lengths were 27.5 cm* and 26.0 cm*, respectively. **B** The BTS on left side shows increased pistoning of stump within the prosthetic socket. **C** The 3D reconstruction reveals a rigid structural frame formed by the plateau on the TFS side

The right lower limb underwent treatment by TFS (Fig. 1A and B) 6. As opposed to the originally described tenodesis, a myoplasty sling was created by attaching the residual Achilles tendon to the remnant of the tibialis anterior muscle tendon distal to the bone bridge (Fig. 1C). Muscle pretension was set to the original resting length, as determined by resting-length markings placed before tendon detachment [11]. The purpose of this tenodesis sling was to facilitate tendon sliding around the bone trochlea during flexion and extension of the residual lower leg muscles, thereby improving proprioceptive feedback (Fig. 1C, Video 1) [12]. Consequently, this procedure requires a slightly longer total stump length (Fig. 2A). In this patient the tibia was osteotomized at 27.2 cm* (*refers to distance from medial tibial plateau to distal stump end throughout the manuscript) to allow for a myoplasty sling with tibialis anterior muscle and tendon. A pedicled fibula bone flap was raised by proximal and distal osteotomy. To achieve the weight bearing bone plateau, the raised fibula flap was transferred horizontally towards the osteotomized tibia, based on its proximal pedicle. Screw fixations were performed to secure the fibula strut in place (3.5 mm cortical screws, DePuy Synthes; *n* = 2, arranged in angulated configuration) (Fig. 2A and C).

On the contralateral side, a conventional BTS approach was constrained by clinical suspicion of chronic osteitis of the fibula [13]. Nevertheless, the total stump length of 27.5 cm* for the tibia (26.0 cm* for the fibula) was chosen equally to the TFS side by request of the patient. The anterior triangular ridge of the tibia was flattened by osteotomy (Fig. 2A). Residual muscle tissue was used for a bulky myodesis to periosteum at the distal stump. Regardless of the respective techniques applied, both surgeries were conducted under tourniquet control, soleus muscles were uniformly resected proximally, nerve stumps were managed by proximal transposition into muscle tissue, remaining sharp edges were smoothed using a bone file and skin closure was accomplished by dorsally based myocutaneous fillet flaps.

### Four Square Step Test (FSST)

The FSST is a clinical test, that has been established to objectively assess dynamic standing balance [14]. The test set up consists of four flat squares on the ground, that are separated by canes in a cross configuration, whereas each cane is 2.5 cm in height and 90.0 cm in length. The task requires subjects to change direction as fast as possible while stepping forward, backward, and sideway (towards right and left), over the cane obstacles without contacting the barrier cross. The sequence starts with the patient standing in square 1 facing square 2, subsequently steps are made forward, sideways, and backwards towards square numbers 2–3 – 4–1 – 4–3 – 2–1. The time from the first foot contact in square 2 to the last foot contact in square 1 is recorded. To detect possible differences between BTS and TFS being either the free or standing leg, the amputee was asked to execute the step backward with the BTS and TFS respectively.

### Timed Up and Go Test (TUGT)

The timed up-and-go (TUG) test evaluates basic functional maneuvers, such as standing up, walking, turning around, and sitting back [15]. In short, the requires the timed rise from a standard armchair, a walk of a 3 m distance, a turn, a walk back, and a sit-down task.

### Gait comfort and clinical assessment

The gait comfort assessment included interviews on subjective laterality preferences in various locomotor tasks, such as prolonged standing phases, circular gait, and stair walking.

### Gait analysis and pressure load distribution

Spatio-temporal walk parameters, force of gait behavior (ground reaction force), as well as dynamics in shifting of weight, center of pressure (COP) trajectory during walk (butterfly), were obtained by treadmill-based electronic gait analysis (C-Mill, Motek, Hocoma, Volketswil, Switzerland). The walking speed was 1.9 km/h with 569 gait cycles and a distance of 314 m. Pressure load distributions in the respective stumps were assessed via a foot pressure measuring system (T&T medilogic, Schoenefeld, Germany). Both residual limbs were fitted with identical prosthetic components by the same prosthetist.

### MR imaging

Imaging was performed on a Siemens Skyra 3 Tesla scanner (MAGNETOM Skyra, Siemens, Erlangen, Germany) with a 20-channel head-neck coil system. Functional images were acquired using a T2-weighted echo planar imaging (EPI) sequence (TR = 3000 ms, TE = 35 ms, flip angle = 90°, FoV = 192 × 192 mm², voxel size = 2 × 2 × 3 mm³, 28 slices). To depict fMRI results on the individual anatomy of the patient, a high-resolution T1-weighted 3D data set (TR = 1910 ms, TE = 3.67 ms, flip angle = 9°, FoV = 256 × 256 mm², voxel size = 1 × 1 × 1 mm³) was additionally obtained. For probabilistic tractography, Diffusion Tensor Imaging (DTI) data were acquired with b-values of 0 and 1000 s/mm^2^ in 30 gradient directions (TR = 5200 ms, TE = 95 ms, FoV = 230 × 230 mm^2^, flip angle = 90°, voxel size = 1.8 × 1.8 × 3 mm^3^).

### fMRI experimental setup

During the fMRI experiment the patient was asked to push his stump repeatedly against a scale held by an investigator. The scale read-out was continuously monitored, and verbal feedback was provided to keep the applied force comparable between the two sides. There were 4 blocks of movement and 4 intermittent resting periods, each lasting 24 s. The task was conducted in separate session for the right and left stump. Movement and resting periods were indicated on a MR-compatible monitor (Cambridge Research Systems, Rochester, UK) at the end of the MR gantry which was reflected via a mirror mounted over the head of the patient. The stimulus presentation (visual indication of movement or resting period) was performed using Presentation (https://www.neurobs.com/). During the movement period the screen turned green with a fixation cross in the middle of the screen, while the screen turned red during resting periods. The patient was instructed to fixate the black cross in the middle of the screen during the whole task. Before scanning, the patient was acquainted with the task outside the MRI scanner.

### fMRI postprocessing and data analysis

For fMRI data analysis, motion correction of functional images and co-registration of the functional images to the anatomical images was performed. No normalization to a standard space or data smoothing was applied. All data analyses were performed using SPM12 (https://www.fil.ion.ucl.ac.uk/spm/). In a first level statistical design two regressors for moving the right and left stump were modeled separately using a 24s long boxcar convolved with a hemodynamic response function. Resting period served as implicit baseline. Using the general linear model, we calculated individual T-maps for the contrasts right and left stump movement versus rest. Also, right and left stump movement was compared against each other. Threshold was set to *p* < 0.001 uncorrected on voxel level and *p* > 0.05 in cluster level (FWE-correction). As is common for single-subject exploratory analyses, no family-wise-error (FWE) or false-discovery-rate (FDR) correction was applied at the voxel level; inference was therefore based on cluster-level statistics, and all fMRI findings are considered exploratory and hypothesis-generating. Anatomical classification was done semi-automatically. Therefore, anatomical regions of interest (ROIs) of the left and right pre- and postcentral gyrus as well as the left and right supplementary motor cortex were extracted from the AAL-atlas (https://www.gin.cnrs.fr/en/tools/aal/) and normalized into the individual space of the patient’s brain [16]. Activation clusters in these ROIs were assigned to the individual anatomical location.

### Probabilistic tractography

Probabilistic tractography was performed using FSL 6.0.3 [17–19]. Anatomic, functional and diffusion data were co-registered using FLIRT [20–22]. For co-registration of DTI and anatomic images, the epi_reg script implemented in FLIRT was used. After brain extraction [23] and correction of eddy-current induced errors and patient movement artifacts [24] as well as susceptibility-induced errors [19, 25], probability models for the distribution of diffusion parameters were calculated using Markov Chain Monte Carlo sampling for each voxel [26, 27]. The matrix of the co-registration between anatomic sequences and DTI images was subsequently applied to the binary activation pattern masks from the fMRI to transfer these ROIs into the common space serving as seed regions for seed-based tractography. Streamlines originating from these regions of interest were calculated on a whole-brain base (cval = 0.2, Sval = 2000, stepl = 0.5, Pval = 5000, fibt = 0.01, distt = 0.0, sampvox = 0.0) resulting in track probability maps [28].

## Results

### Gait comfort

There was no subjective preference regarding laterality towards the BTS or TFS stump for locomotor tasks. The activation of extensors and flexors clinically showed a visible tenodesis-induced movement of the soft tissues on the TFS side. (Video 1 in Supplementary Material). However, the patient reported the TFS stump as his first choice for longer standing phases on both extremities as this caused less back pain during longer stance periods. Furthermore, gait unsteadiness was perceived during loading the BTS side during FSST and circular gait (Video 2 A and 2B, Video 3 A and 3B in Supplementary Material).

### Functional performance

The patient completed all functional assessments. However, less time was needed when the TFS stump was used for stair ascent in a step-to-step gait strategy and to initiate ambulation in the TUGT [29, 30] (Table 1).

**Table 1:** Functional outcomes and gait parameters. Results of clinical functional tests (stair ascent, Timed-Up-and-Go) and treadmill-based spatio-temporal gait analysis Values are given as mean ± SD where applicable. Weight distribution is expressed as absolute axial load in kg with the proportion of total body weight in parentheses (%). “Climbing leg” denotes the leading limb used to ascend the step; “starting leg” denotes the limb used to initiate ambulation in the Timed-Up-and-Go test

	Left	Right
Stance Phase	1.39 s	1.40 s
Single Stance Phase	0.493 s	0.497 s
Step Length	0.518 ± 0.06 m	0.523 ± 0.04 m
Weight Distribution	143 kg (100.5%)	141 kg (99.5%)
Stair ascent (Climbing Leg)	23.17 s	18.72 s
Timed-UP-Go (Starting Leg)	31.39 s	29.74 s
Pressure Load Distribution (mean of a 9 inch sole)	27%	30%

mean ± SD

**Fig. 3:**
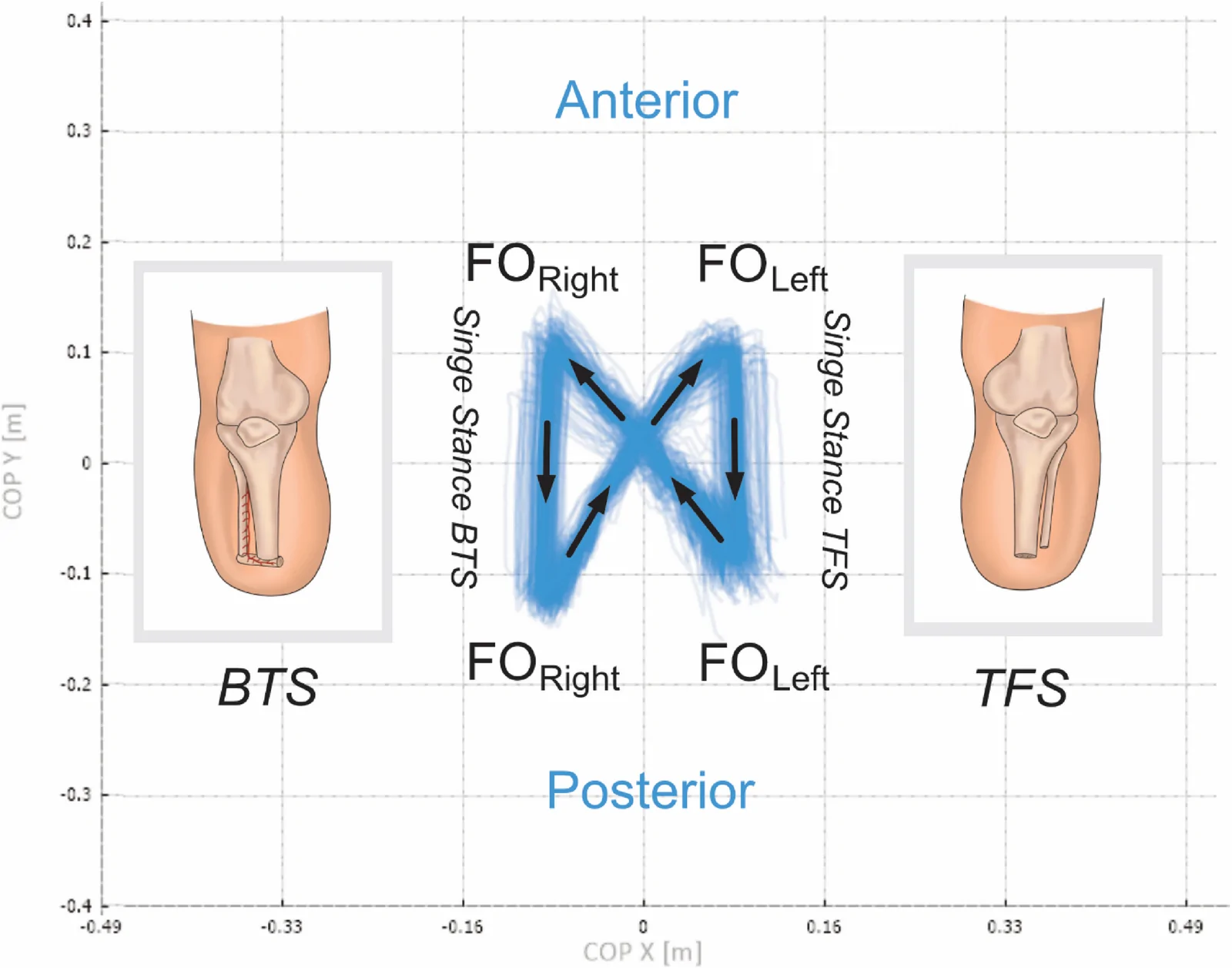
The graphs displays the center of pressure (COP) trajectory during gait. This plot displays displacement of the vertical force vector between the walking patient and the ground surface during multiple gait cycles. A physiologic COP trajectory with medio-lateral and anterior–posterior weight shifts during alternating steps resembles a butterfly shape with a cross symmetry between left and right, as well as with overlapping of single gait cycles to form nearly straight and narrow lines. The diagram provides essential gait characteristics of postural control, such as symmetry, stride width and variability between the extremities. In this patient the COP distribution is asymmetric with a longer relative duration of the single-support stance phase on the BTS leg. A large variability (i.e., a larger array of vector changes) can be detected during the transition from the single support stance phase of the right prosthetic foot and the foot contact of the left leg (FC Left). The double support stance phase from FC Left (foot contact left) to FO Right (foot off right) shows more pressure spread when weight is shifted from right to left. This observation suggests the vertical stump displacement within the prosthetic socket and limited proprioceptive feedback of the BTS. In contrast, vector displacement in double support stance during the stride shift from the left BTS to the right TFS (i.e., from the bottom of the left wing to the top of the right wing) was of relatively longer duration with less vector variability. Left: Foot Contact Left; FO Right: Foot Off Right; FC Right: Foot Contact Right; FO Left: Foot Off Left

Prosthetic treadmill spatio-temporal gait analysis revealed slightly longer stance phases and step lengths for the TFS extremity, whereas total weight distribution was more pronounced on the BTS side (Table 1). Average ground reactions force distribution during loading in the BTS gait cycle presented a delayed increase of force that progressed linearly to a higher overall force peak in comparison to the more homogeneous force distribution of the TFS gait cycle. During the push-off phase the force reached higher peak levels and then progressed to a steeper decrease of force on the BTS side (Fig. 3).

**Fig. 4:**
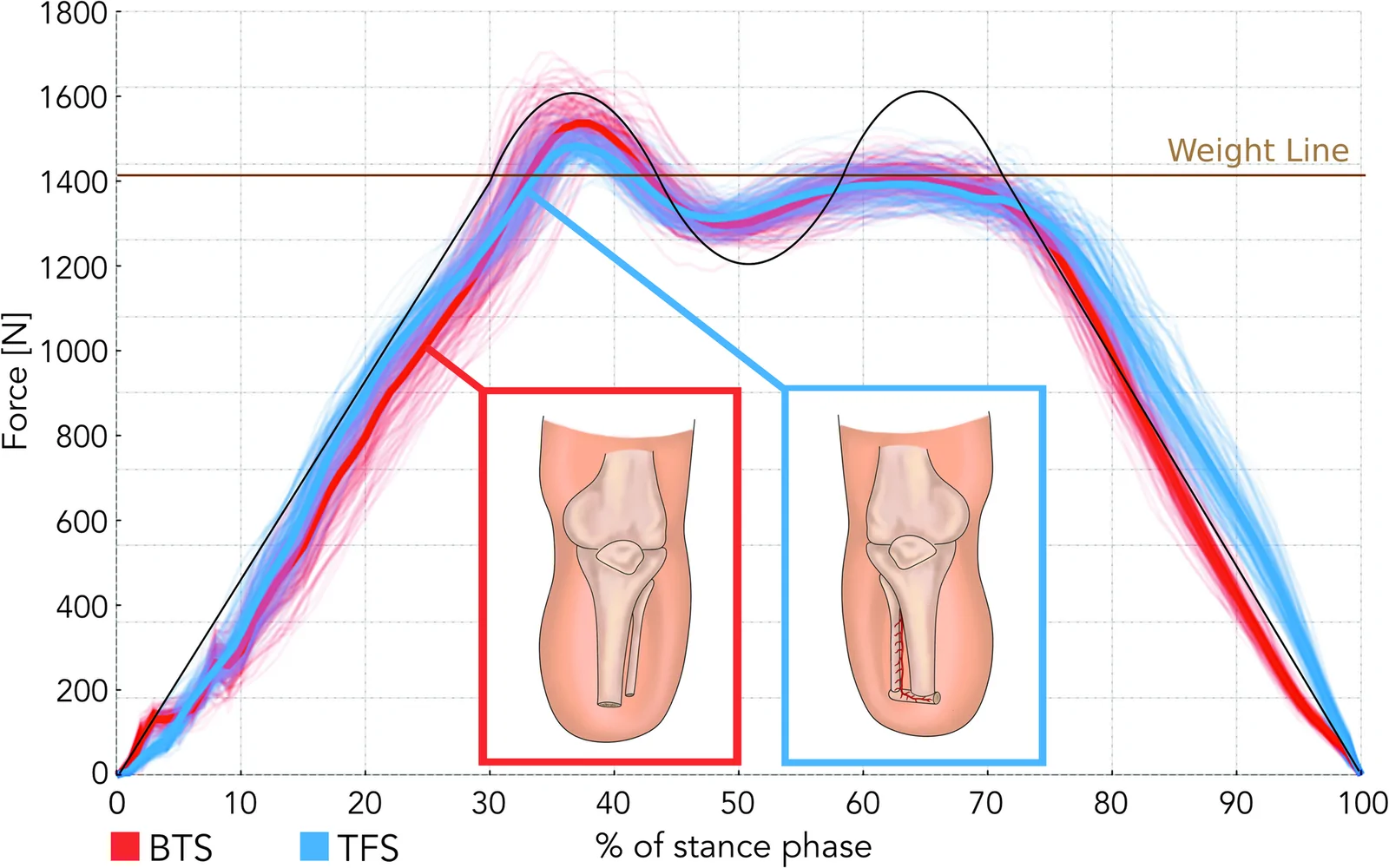
The graphs display vertical ground reaction force during gait. Graphs demonstrate asymmetry of vertical ground reaction force over stance phases of gait between the BTS (red graph) and TFS (blue graph) side. Loading the left BTS stump during stance phases presented a delayed increase of force that progressed linearly to a higher overall force peak during full load (first peak of red graph i.e. heel strike of prosthetic foot). During the push-off phase (second peak of red graph) the force reached higher peak levels and then progressed to a steeper decline of force on the BTS side. Similar prosthetic components on both sides suggests vertical stump displacement within the prosthetic socket as a likely explanation for this observation. In contrast the TFS stump showed a more homogeneous force transition with lower peak pressures during heel loading (first peak of blue graph) as well as during the push off component (second peak of blue graph) together with longer stance phases throughout the gait cycle

The COP distribution showed asymmetry with a longer relative duration of the single-support stance phase on the BTS leg. Large vector variability could be detected during the transition from the single support stance phase of the right prosthetic foot (i.e. TFS) to the double support stance phase. In contrast, vector displacement in double support stance during the stride shift from the left BTS to the right TFS was of relatively longer duration and presented less vector variability (Fig. 4).

Separate pressure distribution analysis of each stump detected maximum pressure values that exceeded 60 N/cm^2^ caused by the tibial component of the BTS, whereas pressure distribution for the TFS stump showed comparably low peak levels with a more homogeneous distribution pattern. (Video 4 A and 4B).

The FSST revealed that the reverse lunge and the sidestep caused test failure by gait unsteadiness and cane contact when the step backward had to be taken with the BTS extremity. For these test circumstances, test failure was recorded for two out of three trials (Video 2 A and 2B). In contrast, when the TFS side was used to overcome the cane barrier, the backstep was larger with no obvious lack of steadiness and without cane contact throughout all repetitions.

### fMRI

**Fig. 5:**
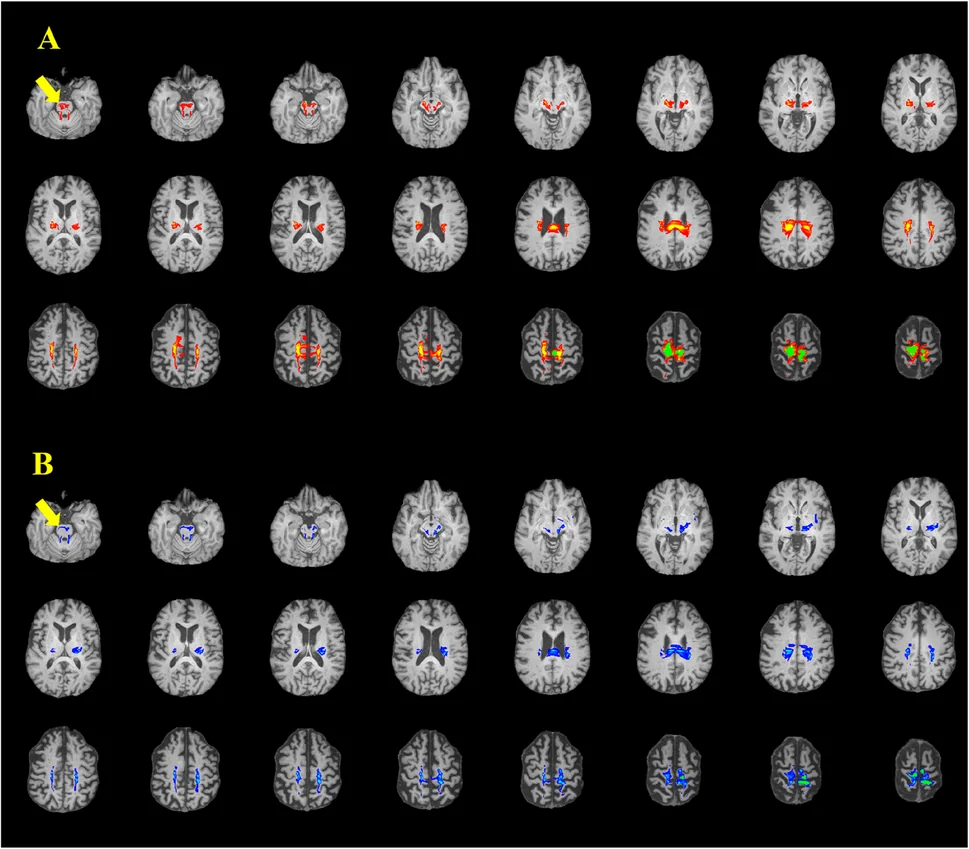
Images of fMRI and DTI Analysis. Results from fMRI and probabilistic tractography when moving the left BTS – stump (**A**) and the right TFS – stump (**B**). Location of fMRI activation maps and fiber tracts are shown on axial T1-weighted images. Green: fMRI activation maps, red: fibers depicted by probabilistic tractography using the fMRI activation map during movement of the left stump as a seed region, blue: fibers depicted by probabilistic tractography using the fMRI activation map during movement of the right stump as a seed region. Yellow arrows indicate partially crossing left-hemispheric fibers at the level of the SCP

Stump loading with equal pressure on the left (TFS) and right stump (BTS) showed bilateral activated clusters in the pre- and postcentral gyrus, as well as in the supplementary motor area ( (*p* = 0.05, FEW corrected; T = 3.13; Table 2) with one exception. There were no activated voxels in the left postcentral gyrus during loading of the left stump Of note, the primary (contralateral) response for left-stump (BTS) loading would be expected in the right postcentral gyrus; a small right-postcentral cluster was detected but did not reach cluster-level significance (Table 2), whereas the contralateral (left) postcentral activation during right-stump (TFS) loading was more pronounced. The absence of ipsilateral (left) postcentral activation during left-stump loading was expected. However, failure to detect activation does not prove the absence of activation and may reflect limited statistical power, signal-to-noise ratio and thresholding effects (Fig. 5). Taken a voxel threshold of *p* < 0.001, those results were statistically significant on cluster level for the left and right precentral gyrus as well as the left supplementary motor cortex when moving the right stump. During movement of the left stump, a significant associated activation was found on cluster level in the left and right supplementary cortex.

**Table 2:** Activated clusters during movement of the right and left stump in the pre- and postcentral gyrus, as well as in the supplementary motor area. (*p* < 0.05 FWE corrected on cluster level, T = 3.13). Abbreviation: Supplementary motor area (SMA)

Location	Cluster size	Z-value on peak level	*p*-value on cluster level
*Moving TFS (right stump)*			
* Left precentral Gyrus + SMA*	*217*	*5.05*	*< 0.001*
* Left postcentral Gyrus*	*10*	*4.76*	*0.189*
* Right precentral Gyrus*	*18*	*4.01*	*0.003*
* Right postcentral Gyrus*	*14*	*4.18*	*0.122*
* Right SMA*	*18*	*4.01*	*0.083*
*Moving BTS (left stump)*			
* Left precentral Gyrus*			
* Cluster 1*	*15*	*3.84*	*0.11*
* Cluster 2*	*4*	*3.38*	*0.401*
* Left postcentral Gyrus*	*-*	*-*	*-*
* Left SMA*	*75*	*4.34*	*0.002*
* Right precentral Gyrus*	*12*	*4.73*	*0.15*
* Right postcentral Gyrus*	*15*	*3.64*	*0.11*
* Right SMA*	*260*	*5.22*	*< 0.001*

Both movement of either the left or right stump showed associated movement-induced activation, which is more pronounced in the contralateral hemisphere. The direct statistical comparison of movement of the left and right stump did not yield any significant differences, probably due to low effect sizes. Qualitatively, moving the right stump is associated with larger voxel clusters in the contralateral precentral gyrus, moving the left stump with larger clusters in the contralateral Supplementary motor area (SMA) (Figs. 7 and 8).

### Probabilistic tractography

fMRI activation clusters during movement of the left stump (TFS) were located more anteriorly compared to those during movement of the right stump (BTS). Probabilistic tractography using these activation clusters as seed regions depicted both pyramidal tracts with interhemispherically crossing fibers (Fig. 5). Additionally, fibers connecting the cortical activation maps with the cerebellum through the Superior cerebellar peduncle (SCP) were found bilaterally. Based on activations maps during movement of the left stump, more extensive streamline reconstructions were obtained, probably due to bigger activation maps compared to those during movement of the right stump (Figs. 7 and 8). Interestingly, left-hemispheric fibers partially crossed at the level of the SCP, which could not be retraced for right-hemispheric fibers, especially during movement of the right stump.

A detailed mechanistic interpretation of these circuits is provided in the Discussion.

Spontaneous voluntary movements are initiated by the primary motor cortex (orange) and basal ganglia (purple), while the supplementary motor area (SMA, blue) plays a key role in planning complex, consciously controlled movements. The SMA also contributes to postural stabilization and the control of internally generated motion. Compared to the contralateral stump, movement of the left stump showed larger activation clusters in the contralateral SMA. The cerebro-cerebellar network regulates balance and posture during locomotion. The cerebellum receives proprioceptive input mainly via contralateral spinocerebellar tracts (green circuit), while cerebellar output to the midbrain, primarily via the dentato-rubro-thalamic tract, is transmitted through the superior cerebellar peduncle (SCP; blue circuits). Discrepancies between cortical motor commands and proprioceptive feedback are corrected through cerebellar efferents projecting via the red nucleus (red) to maintain stable locomotion. In this patient, probabilistic tractography using fMRI activation clusters revealed the pyramidal tract (red circuit), cerebro-cerebellar, and cerebellar-cerebral pathways running bilaterally through the SCP. Stimulation of the left BTS stump produced larger activation maps and more extensive streamline reconstructions, possibly reflecting adaptive plasticity due to reduced interoceptive input (dotted green line) from the left BTS stump.

## Discussion

At least in theory, there are several aspects that suggest more favourable physiological properties of the TFS approach. The bone bridge enlarges the bony stump area and thereby increases potential weight bearing properties of the residual limb. Accordingly, the comparison of pressure load distributions between TFS and BTS stump indicated that local pressure peaks at the tibial component of the BTS stump exceeded the detected forces of the TFS arrangement (Video 4 A and 4B). The bone plateau has the potential to improve skin to interface ambulatory pressure distribution. Moreover, the prosthetic x-ray together with the assessment of vertical displacement during stance suggested, that the bone strut might considerably limit vertical pistoning of the stump during axial load transmission within the prosthetic socket [31] (Fig. 2B). However, ambulation characteristics suggested, that this decrease of pistoning in the TFS stump may as well translate into improved mechanical advantage and consequently increased force efficiency during gait. The prosthetic gaitogram revealed COP asymmetry in the butterfly plot, with longer swing phases for the BTS side than for the TFS stump (Fig. 3). Previous assessments of prosthetic ambulation patterns in unilateral trans-tibial amputees determined gaitcycles as commonly asymmetric, with predominantly longer swing phases on the prosthetic side [32]. Regarding the bilateral stump conditions in this amputee, the TFS side seemed to be the intuitive preference for loading during double-support stance phases, as reflected by the prolonged stance phases in the temporal gait analysis (Fig. 4). Of note, despite these extended stance phases of the TFS side, the spatio gait assessment still yielded slightly longer average step lengths for the TFS extremity. Considering the more homogeneous pressure distribution in the right gait cycle (i.e. TFS), together with the detected reduction in socket pistoning, the TFS stump appeared to be of greater efficiency for ambulation in this direct comparison (Figs. 2B and 4). This observation is in line with other investigations, that measured more recruitable hip and knee power while negotiation of curbs and sit-to-stand tasks in TFS compared to BTS cohorts [4, 8]. Likewise, speed performance during stair ascent in a step-to-step strategy, was superior when the patient exclusively used the TFS side to climb the step in this particular task (Table 1).

In TFS stumps, the distal osteosynthesis between the tibia and fibula produces a rigid frame that much more resembles the anatomy of the original ankle mortice (Figs. 1C and 2C). In contrast, the BTS allows purely unconstrained translational and rotational forces between the tibia and fibula stump [33] (Figs. 1C and 2C,´). Although, this instability between the bone stumps is likely to be more pronounced in circular walking as required for the FSST, increased step lengths in the gait cycle might as well be a consequence of the greater anatomic stability of the TFS extremity [4, 34] (Fig. 3).

Trans-tibial stumps less than 12.5 cm* in length may gradually decrease leverage within the socket, especially during transverse loading, which deteriorates optimal prosthetic fitting, reduces mechanical advantage, and furthermore has been suggested to negatively affect postural control [34–36].

Another asset of a TFS, is its potential to provide a smooth but rigid trochlea for unconstrained tendon motion (Fig. 1C and Video 1). Proprioceptors, within the muscle tissue and periosteum constantly detect kinematic parameters and trigger reflexes at spinal cord level as well as at the cortical, thalamic and pontocerebellar level, that play an integral part of the sensorimotor control and related coordinated locomotion [12, 37–39] (Figs. 7 and 8).

**Fig. 6:**
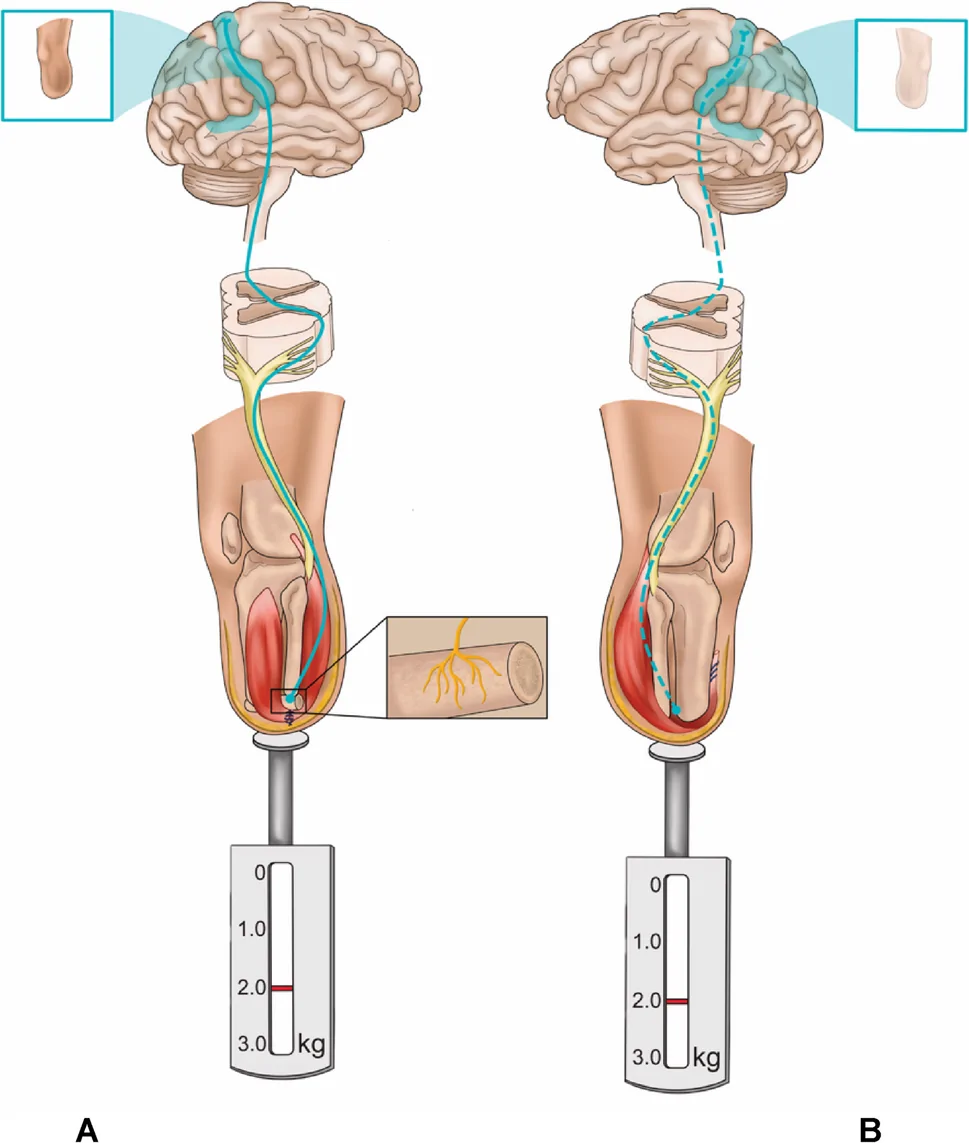
Illustrations of fMRI circuits during stump loading. **A** and **B** Stump loading with equal pressure on both sides during fMRI. **B** There was no activation in the left postcentral gyrus (light blue) during loading of the left BTS stump. The findings suggest a lack of stimulus response (dotted light blue circuit) on the left side – possibly reflecting enhanced proprioceptive feedback via the enlarged surface of the bony TFS plateau

Lower-limb amputees are more susceptible to falls, predominantly during stair ascent [40]. However, the distal connection of plantar extensor and plantar flexor muscles reclaims proprioceptive feedback, by moving about this trochlea with simultaneous excursion of the respective muscle fibers throughout contraction and extension within stride as well as stance phases (Fig. 1C and Video 1). Without this reconstructive effort, remaining proximal proprioceptors and the vestibular system alone fail to sufficiently alleviate the loss of distal proprioception in lower limb amputees [36, 40, 41] (Fig. 6A and B). Only recently, Butowicz et al. astutely identified vision as the primary source of balance in lower leg amputees [42]. Moreover, the FSST entails stepping backwards across the cane barrier without visual feedback for the subject. When this patient was instructed to take this specific step with the BTS extremity first, it led to reproducible gait unsteadiness with evasive steps and even test failure upon touching the barrier (Video 2 A and 2B).

**Fig. 7:**
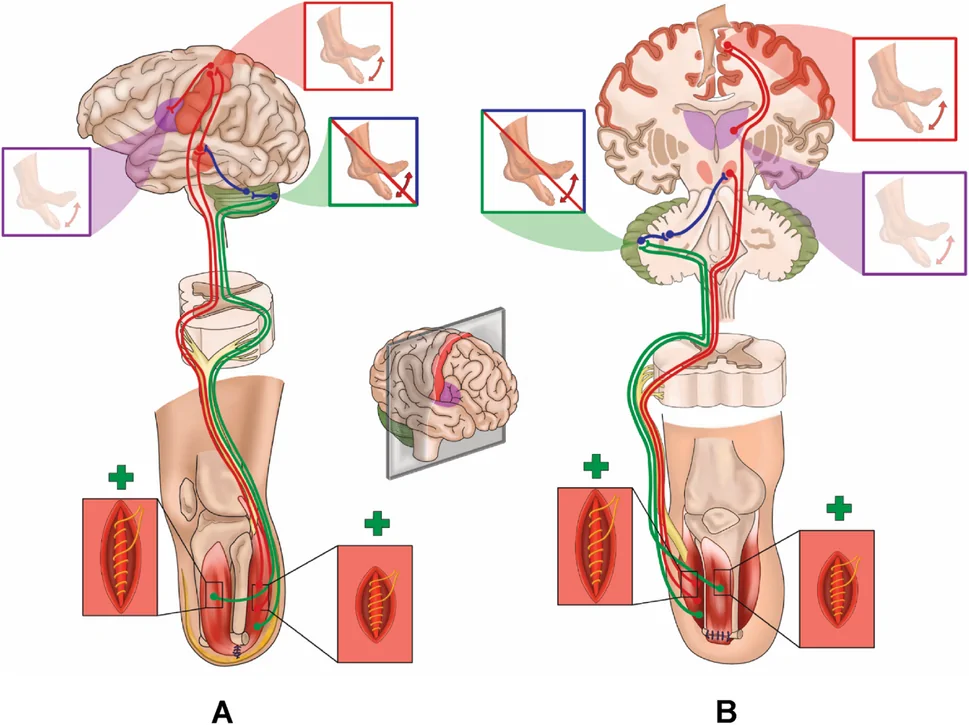
Illustrations of fMRI circuits during motion of right stump (A side view, B cross section). Spontaneous voluntary movements are initiated by the primary motor cortex (orange) and the basal ganglia (purple). Qualitative analysis revealed larger voxel clusters in the contralateral primary motor cortex during movement of the right stump, whereas motion of the left stump was associated with larger clusters in the contralateral supplementary motor area, as shown in Fig. 8

These neuroplastic processes were primarily observed in the postcentral somatosensory cortex, resulting in an increased interhemispheric asymmetry regarding body maps in this brain area [43, 44]. Alternating axial loading on both stumps during fMRI resulted in a significant activation of the contralateral postcentral gyrus exclusively for stimulation of the TFS stump. Likely, the unaltered feedback by the rigid bony plateau together with proprioceptive features of the tendon sling are causative for this observation (Fig. 7).

**Fig. 8:**
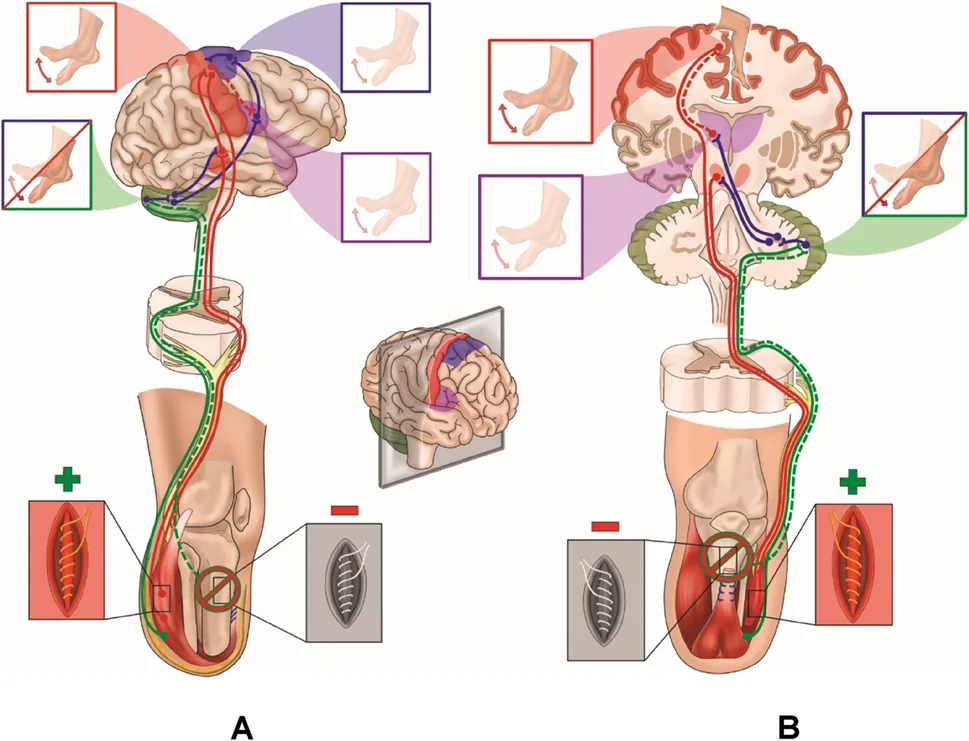
Illustrations of fMRI circuits during motion of left stump (**A** side view, **B** cross section)

Although, not yet fully explored, functions attributed to the SMA are postural stabilization of the body, coordination between contralateral sides during motor tasks and control of internally generated movements [45]. Additionally, the premotor cortex as well as the basal ganglia, forming a functional circuit between the thalamus and the motor cortex play a role in the execution and modulation of movements [46, 47] (Fig. 8).

In this patient, fMRI revealed significant SMA activation predominantly during voluntary movement of the left phantom limb with the BTS. We suspect, that the enhanced interoceptive feedback provided by the tendon sling of the TFS stump reduces the discrepancy arising from the comparison between the predicted and actual sensory feedback, consequently necessitating less activation of the SMA (Fig. 8). In contrast to the BTS, the straight opposition of agonistic and antagonistic receptors within the tendon sling of the TFS have the potential to transform any motor command into an immediate sensory consequence - similar to the interoceptive feedback of a non-amputated limb [48]. This hypothesis is consistent with studies that identified activation of SMA for the voluntary movement of phantom limbs in amputees, but not for non-amputated extremities [49].

Cerebellar efferences to the midbrain, mainly represented by the dentato-rubro-thalamic tract, are carried by the SCP [50]. In case of discrepancies between motor commands executed by the cerebral cortex and sensory responses, these cerebellar efferences rectify errors to maintain stable locomotion [51] (Figs. 7 and 8). Within the context of lower limb amputees, a preceding study found a decrease of grey matter volume bilaterally in the cerebellum in patients which never used a prosthesis, but not in amputees who do use prothesis, suggesting an influence of prosthetic care in these neuroplastic changes [44].

Probabilistic tractography in this patient using the activation clusters from fMRI detected both the pyramidal tract as well as cerebro-cerebellar as well as the cerebellar-cerebral fibers running through the SCP bilaterally. As stimulation of the left BTS stump led to bigger activation maps, the streamline reconstructions associated with this side were more extensive. This might be the consequence of an adaption process due to the lack of interoceptive input from the left BTS stump (Fig. 8).

Fibers originating from the left dominant hemisphere partially crossed in the brain stem. These fibers could be tractographically retraced when using both the fMRI activation maps during stimulation of the left as well as the right stump. Of course, the depiction of these fibers could also be artificial and only be linked to methodological pitfalls associated with tractographic approaches.

Among the greatest disadvantages of the TFS procedure is the prolonged rehabilitation process because the bone fixation does not provide immediate stability. This is especially relevant in geriatric patients, that bear a constant risk of deterioration of internal condition and physical constitution during the phase of immobility. Of note, existing data does not suggest a significant increase in complications rates for TFS in elderly populations [52]. Still, most optimal candidates for TFS are most likely high-performing individuals that seek vigorous post-amputation activity. A retrospective trial in US service members confirmed the ability for post-amputation deployment in 13% of TFS patients, versus 7% of BTS amputees [53]. Therefore, most reports are still unable to provide robust evidence to guide clinical practice [54].

Although, this trial attempted to emphasize potential strengths and weaknesses of these procedures from a highly undistorted individual perspective, it is within the nature of this pre-defined trial set up, that the authors were unable to obtain sufficient quality of life data. Existing questionnaires are commonly designed for unilateral amputees, which excludes closer insights on the specific impact of either stump on quality of life. Another uncertainty might include the time lag of several months between TFS and BTS surgeries, which may have influenced individual habits in respect to weight loading and spatio-temporal parameters. Still, objective stump characteristics determined high consistency with assessed functional performances, and thereby supported the two main hypothesis behind TFS stump management: Improved force distribution during pressure load and increases efficiency of force transmission during prosthetic gait. The inter-surgical interval of six months, and the associated differences in prosthetic experience, motor adaptation and possible cortical plasticity between the earlier-operated (TFS) and later-operated (BTS) side, represent a potential confounder that may have influenced both the functional and the neuroimaging findings. Furthermore, it is impossible to precisely determine, whether the observed effects resulted from the bone management (TFS vs. BTS) or the soft-tissue reconstruction (tendon sling vs. conventional myodesis) or a combination of both. Additional limitations of this single-case report include the lack of validated patient-reported outcome measures and the short-term nature of the follow-up. The FSST was based on three trials assessed by a single examiner, which precluded formal analysis of repeatability and reliability. The fMRI results were thresholded at *p* < 0.001 uncorrected without FWE or FDR correction and are therefore exploratory, and DTI tractography provides probabilistic streamline estimates rather than a direct measure of connectivity strength. Accordingly, statements regarding enhanced proprioception, improved feedback and greater efficiency throughout this report should be understood as hypotheses generated by this individual case.

Furthermore, the patient may have benefited from the restoration of the antagonistic muscle stretch through putatively improved proprioceptive feedback, particularly in situation of impaired visional feedback and stair ascent (Video 2 A and 2B). Although, the investigated patient met the criteria of effective daylong ambulation and routine participation in recreational activities – and some sports, the BMI does not necessarily imply high athletic demands. Perhaps, the determined differences in stump performance of this investigation may be even more pronounced in athletically ambitious individuals, such as e.g. active service members.

## Conclusion

In direct comparison to a conventional stump, a TFS approach in conjunction with a tenodesis sling appeared to translate into a favourable functional outcome in this within-subject comparison. Possibly, improved proprioceptive awareness may mitigate the loss of kinesthesia as otherwise experienced by lower limb amputees, whereas the bone plateau may have reduced stump pistoning and related loss of mechanical advantage.

The undistorted insights of this trial may facilitate preoperative counseling by data driven arguments, when it comes to the individual needs of a specific patient.

Despite the advances of modern microprocessor driven prosthetic systems, progress towards more functional as well as individual aspects in surgical stump management must not be disregarded.

## Supplementary Information

Supplementary Material 1: Video 1: Stump motionActivation of the extensors and flexors produced a clear tenodesis effect of the soft tissues on the TFS side, whereas the BTS side showed minimal to no excursion. Voluntary flexion and extension induces opposing contraction / stretch in the gastrocnemius and the tibialis anterior muscle and vice versa on the TFS side but not on the BTS side.

Supplementary Material 2: Video 2: Four Square Step Test (FSST) (A right TFS, B left BTS)Reverse lunge and sidestep executed with TFS and BTS respectively in the FSST. The use of the BTS was associated repeatedly with test failure by gait unsteadiness and cane contact during the step backwards.

Supplementary Material 3: Video 3: Circular Walk (A Circular Walk Right Orientation, B Circular Walk Left Orientation)Loading of the BTS side was perceived as gait instability during circular walking in a leftward direction.

Supplementary Material 4: Video 3: Circular Walk (A Circular Walk Right Orientation, B Circular Walk Left Orientation)Loading of the BTS side was perceived as gait instability during circular walking in a leftward direction.

Supplementary Material 5: Video 4: Pressure load distribution of stumps (A right TFS, B left BTS)Videos demonstrate pressure distribution analysis of respective stumps. The left side of the screen shows the heat map of the pressure load through the stump. The center of the image shows the CT reconstruction of the stump. The right side of the image shows the loaded residual limb surface and the pressure curve in N/cm2. A Peak pressure of the TFS stump remained comparably low and presented a homogeneous distribution pattern. B Loading of the BTS produced more localized pressure by the tibial component, which was perceived as painful by the patient. Maximum pressure values exceeded 60 N/cm2.

Supplementary Material 6: Video 4: Pressure load distribution of stumps (A right TFS, B left BTS)Videos demonstrate pressure distribution analysis of respective stumps. The left side of the screen shows the heat map of the pressure load through the stump. The center of the image shows the CT reconstruction of the stump. The right side of the image shows the loaded residual limb surface and the pressure curve in N/cm2. A Peak pressure of the TFS stump remained comparably low and presented a homogeneous distribution pattern. B Loading of the BTS produced more localized pressure by the tibial component, which was perceived as painful by the patient. Maximum pressure values exceeded 60 N/cm2.

## Data Availability

All data can be made available upon request by contacting the corresponding author.

## Abbreviations

TFS: tibiofibular synostosis
BTS: Burgess-type stump
COP: Centre of pressure
FSST: Four Square Step Test
TUGT: Timed-Up-and-Go Test
fMRI: Functional magnetic resonance imaging
DTI: Diffusion tensor imaging
EPI: Echo planar imaging
ROI: Region of interest
AAL: Automated anatomical labelling
SMA: Supplementary motor area
SCP: Superior cerebellar peduncle
FWE: Family-wise error
FDR: False discovery rate
PROM: Patient-reported outcome measure
BMI: Body mass index
